# DCLK1 Monoclonal Antibody-Based CAR-T Cells as a Novel Treatment Strategy against Human Colorectal Cancers

**DOI:** 10.3390/cancers12010054

**Published:** 2019-12-23

**Authors:** Sripathi M. Sureban, Robert Berahovich, Hua Zhou, Shirley Xu, Lijun Wu, Kai Ding, Randal May, Dongfeng Qu, Edwin Bannerman-Menson, Vita Golubovskaya, Courtney W. Houchen

**Affiliations:** 1 COARE Holdings Inc., Oklahoma, OK 73104, USA; randal-may@ouhsc.edu (R.M.); dongfeng-qu@ouhsc.edu (D.Q.); eddie@bannermanmenson.com (E.B.-M.); 2 Department of Internal Medicine, Digestive Diseases and Nutrition Section, The University of Oklahoma Health Science Center, Oklahoma, OK 73014, USA; kai-ding@ouhsc.edu; 3 ProMab Biotechnologies Inc., Richmond, CA 94806, USA; Robert.berahovich@promab.com (R.B.); huazhou369@gmail.com (H.Z.); shirley.xu@promab.com (S.X.); john@promab.com (L.W.); vita.gol@promab.com (V.G.); 4 Veterans Affairs Medical Center, Oklahoma, OK 73104, USA

**Keywords:** DCLK1, CAR-T, tumor stem cells, immunotherapy, clonogenicity

## Abstract

CAR-T (chimeric antigen receptor T cells) immunotherapy is effective in many hematological cancers; however, efficacy in solid tumors is disappointing. Doublecortin-like kinase 1 (DCLK1) labels tumor stem cells (TSCs) in genetic mouse models of colorectal cancer (CRC). Here, we describe a novel CAR-T targeting DCLK1 (CBT-511; with our proprietary DCLK1 single-chain antibody variable fragment) as a treatment strategy to eradicate CRC TSCs. The cell surface expression of DCLK1 and cytotoxicity of CBT-511 were assessed in CRC cells (HT29, HCT116, and LoVo). LoVo-derived tumor xenografts in NOD Scid gamma (NSG™) mice were treated with CBT-511 or mock CAR-T cells. Adherent CRC cells express surface DCLK1 (two-dimensional, 2D). A 4.5-fold increase in surface DCLK1 was observed when HT29 cells were grown as spheroids (three-dimensional, 3D). CBT-511 induced cytotoxicity (2D; *p* < 0.0001), and increased Interferon gamma (IFN-γ) release in CRC cells (2D) compared to mock CAR-T (*p* < 0.0001). Moreover, an even greater increase in IFN-γ release was observed when cells were grown in 3D. CBT-511 reduced tumor growth by approximately 50 percent compared to mock CAR-T. These data suggest that CRC cells with increased clonogenic capacity express increased surface DCLK1. A DCLK1-targeted CAR-T can induce cytotoxicity in vitro and inhibit xenograft growth in vivo.

## 1. Introduction

Colorectal cancer (CRC) is the third leading cause of cancer-related deaths in the U.S. [1]. For individuals diagnosed with stage IV of the disease, the five-year survival rate is just 12% [1]. A growing body of evidence suggests that cancer stem cells (CSCs) play a critical role in the initiation, progression, and metastatic spread of cancer [2,3]. In solid tumor cancers, including CRCs, the tumor attempts to either evade the anti-tumor immune system or emit inhibitory signals to suppress immune effector cells and limit their anti-tumorigenic activity [4,5].

Immunotherapy has been remarkably successful for treating solid tumor cancers due to its unique ability to inhibit immune system checkpoint proteins (e.g., PD-1, PD-L1, CTLA4) and reactivate the host’s anti-tumor immunity [6]. Mechanistically, these checkpoint inhibitors enable cytotoxic CD8^+^ T cells to attack and destroy cancer cells [6]. However, the efficacy of these inhibitors as a monotherapy or in combination with other therapeutics varies considerably and is largely dependent on tumor type and individual patient characteristics [7]. In addition, the composition, pH, and metabolic activity within the tumor microenvironment (TME) are additional factors that can influence the effectiveness of these agents [8]. Infiltrating T lymphocytes that invade the TME and exert cytotoxic effects within the tumor have been shown to correlate with increased efficacy and improved survival rate in several cancers [8,9]. However, the efficacy again varies greatly between patient populations and the type of tumor being targeted. 

The CSC hypothesis is based on findings that solid tumor cancers, including CRCs, are derived from a rare population of long lived, relatively quiescent, self-renewing cancer cells that are able to differentiate into every individual epithelial cell type within the tumor mass [10,11]. These cells are also able to differentiate into non-epithelial cell types via a process called epithelial-mesenchymal transition (EMT) [3,12,13,14,15,16]. This process facilitates TSC entry into the blood stream prior to dissemination and uptake into distant organs where they re-epithelialized through a process known as mesenchymal-epithelial transition (MET) [17]. Although the molecular mechanisms that regulate these highly integrative processes are under investigation, their contribution to advanced CRC-related mortality is clear. Inhibition of protooncogenic processes within the TSCs, as well as TSC eradication and inactivation, are crucial for successful anti-cancer treatments [10,11,18]. Recent studies have demonstrated that increased expression of EMT transcription factors and CSC-related proteins in human CRC tissues are strongly associated with unfavorable clinicopathological manifestations and poor patient outcomes [18]. Given the aggressive nature of CRC tumors and their high rate(s) of recurrence or metastasis, EMT-and CSC-related proteins may provide novel therapeutic targets for the treatment of CRC [10,11,18].

Doublecortin-like kinase 1 (DCLK1) is a microtubule-associated kinase that regulates EMT and is associated with microRNAs known to regulate tumor growth and progression [19,20,21,22,23,24]. A compelling study published in Nature Genetics utilizing the Dclk1^CreErt2^ mouse model demonstrated that Dclk1 specifically marks TSCs that continuously produce tumor progeny in the polyps of *Apc^Min/+^* mice. In addition, specific ablation of Dclk1^+^ TSCs resulted in marked polyp regression and no intestinal damage [25]. This landmark study was the first to identify Dclk1 as a marker that can distinguish normal gastrointestinal epithelial cells from TSCs and to demonstrate that normal intestinal Dclk1 expressing epithelial cells are not required for normal homeostatic function [25]. In another study, quiescent Dclk1^+^ tuft cells served as colon cancer-initiating cells following loss of *Apc* and in the presence of inflammation in a *Dclk1^CreErt^* mouse model [26]. In human CRC tumors, elevated levels of DCLK1 are associated with higher rates of recurrence and mortality [27]. Therefore, strategies directed at eliminating DCLK1-expressing TSCs have the potential to mitigate CRC-related morbidity, recurrence, and metastasis and improve survival in patients afflicted by this insidious disease. 

Immunotherapy using CAR-T (T cells modified with chimeric antigen receptor) is recognized as an increasingly effective therapy for the treatment of hematologic malignancies [28,29,30]. However, its efficacy in treating solid tumor malignancies has been less promising [31]. Several hypotheses have been generated that may explain this treatment disparity: a hypoxic TME that reduces CD8^+^ cytotoxic T cell viability, tumor associated induction of innate immunosuppression, and solid tumor-based physical impediments that prevent T cell cytotoxicity against the tumor [32,33,34,35]. These solid tumor-associated features are not encountered during systemic administration of CAR-T therapies used for hematologic malignancies [32,33,34,35]. CAR-T therapy is an exciting new treatment modality in which a patient’s CD8^+^ T cell population is removed and re-engineered to create a new population of chimeric T cells. The cells are designed to include an extracellular antigen-binding domain targeting tumor-specific antigens expressed on the surface of cancer cells [36,37]. This personalized approach to cancer therapy enables a patient’s own T cells to be programmed to attack and eliminate their specific cancer. Typically, the tumor antigen specific targeting region is a single-chain antibody variable fragment (ScFv) fused to a hinge, transmembrane domain, and co-stimulatory domains (CD28, 4-1BB, CD27 or others) to stimulate the immune response, as well as a CD3ζ activation domain [38,39,40,41,42,43].

In this report, we demonstrate that the proprietary humanized DCLK1 ScFv sequence can be used to detect cell surface expression of the extracellular DCLK1 (human isoforms 2 and 4) on several CRC cell lines. Furthermore, we demonstrate that HT29 cells grown in three-dimensional (3D) matrices exhibit a 4.5-fold increase in cell surface DCLK1 expression compared to cells grown in two-dimensional (2D). These data support our hypothesis that the TSC population can be targeted using a DCLK1-specific CAR-T. Here, we report that, in collaboration with ProMab Inc., we have developed a novel CAR-T based on the DCLK1 ScFv containing a CD28 transmembrane and co-stimulatory domain and CD3ζ activation domain [39,40,41,42,43,44,45,46]. CAR-T cells generated using DCLK1 ScFv (CBT-511) demonstrated ~20% CAR expression and significantly induced CRC cell cytotoxicity. Compared to mock CAR-T treatments, CBT-511 significantly induced Interferon gamma (IFN-γ) production in CRC cells grown in 2D and 3D matrices, indicating that the CAR-T cells are able to successfully bind/interact with CRC cells (HT29, HCT116, and LoVo). Finally, CBT-511 treatment resulted in significant inhibition of LoVo CRC cells-derived tumor xenograft growth in NSG™ mice. These data taken together strongly suggest that DCLK1 CAR-T can be developed to specifically target TSCs in CRC and perhaps other solid tumors. This paper represents the first demonstration of a DCLK1-directed CAR-T formulation.

## 2. Results

### 2.1. DCLK1 mAb CBT-15 Has High Binding Affinity to DCLK1

Of the four DCLK1 isoforms (1–4), isoforms 2 and 4 are highly upregulated in cancers, whereas isoforms 1 and 3 are not [47,48,49]. Our proprietary DCLK1 mAb, CBT-15, recognizes a 13 amino acid sequence (extracellular domain) of the tumor-specific DCLK1 isoforms 2 and 4 with a binding affinity of < 1 nM Kd. We previously reported that targeting renal cell carcinoma TSCs with CBT-15 resulted in xenograft growth arrest and EMT inhibition [47]. Here, we have generated a humanized version of CBT-15 (hCBT-15) and demonstrated its ability to recognize the extracellular domain of DCLK1. Using flow cytometry, we analyzed several cancer cell lines with hCBT-15. 

**Figure 1:**
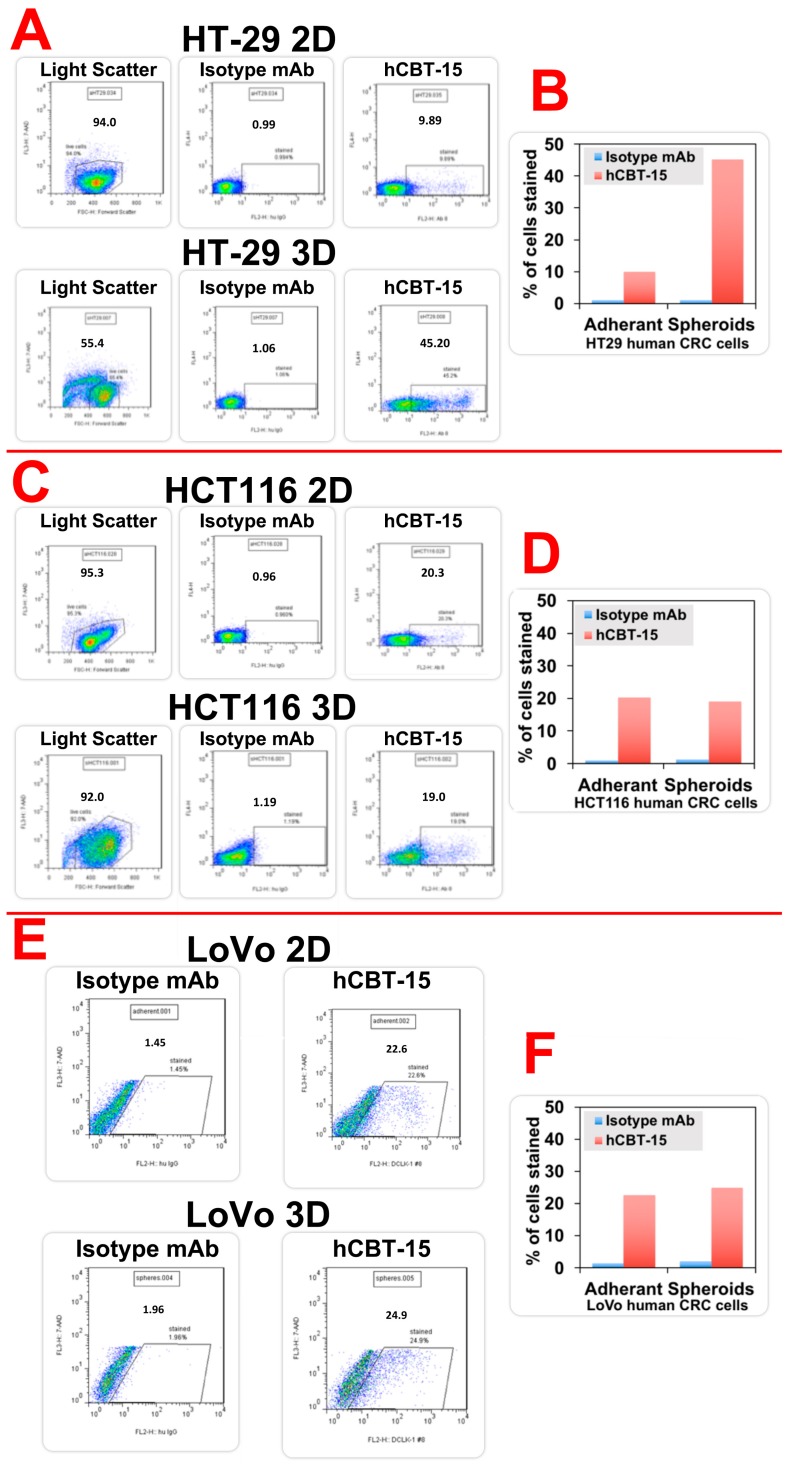
Doublecortin-like kinase 1 (DCLK1) surface expression in human colorectal cancer cell lines. HT29, human colorectal cancer (CRC) cells were grown in adherent 2D tissue culture plates and as 3D spheroids. These cells were disassociated and subjected to Fluorescence-activated cell sorting (FACS) using humanized DCLK1 mAb (hCBT-15). HT29 cells grown in 2D culture demonstrated extracellular DCLK1 expression on 9.89% of cells (**A**—Top panel). HT29 cells grown in 3D matrices demonstrated extracellular DCLK1 expression on 45% of cells (**A**—bottom panel). The quantitative expression is represented in the bar graph (**B**). (**C**,**D**): HCT116, grown in 2D demonstrated DCLK1 extracellular expression on 20% and 3D demonstrated DCLK1 extracellular on 19.5% of cells. (**E**,**F**): LoVo, grown in 2D demonstrated DCLK1 extracellular expression on 22% of cells and 3D demonstrated DCLK1 extracellular on 25% of cells.

When HT29 human CRC cells were grown in 2D matrices, approximately 9% of the total population were positive for surface DCLK1 (Figure 1A). However, when grown in 3D matrices, the HT29 cells developed a spheroid-like morphology and cell sorting revealed a striking increase in surface DCLK1 to 45% of the cells (Figure 1A,B). Interestingly, when HCT-116 and LoVo cells when grown in 2D matrices, we observed 20% and 22% of cells expressing surface DCLK1, respectively. However, when grown in 3D matrices, no significant increase in cell surface DCLK1 expression was observed (Figure 1C–F). These data taken together suggest that HT29 cells with high clonogenic capacity express a marked increase in cell surface DCLK1 expression. This is not surprising, as DCLK1 marks TSCs in intestinal adenomas and targeting them resulted in complete abrogation of the adenomas [25]. Furthermore, this also provides a strong rationale to utilize the cell surface expression of DCLK1 to deliver anti-cancer payloads or for developing DCLK1-based CAR-T therapy. Interestingly, we did not observe any increases in cell surface expression in HCT116 or LoVo cells when grown in 3D. Although this finding was unsuspected, those two cell lines exhibited approximately two-fold more cell surface DCLK1 than HT29 cells grown on adherent slides.

### 2.2. DCLK1 Is Upregulated in Human CRC and CBT-15 Specifically Recognizes DCLK1 in CRC 

**Figure 2:**
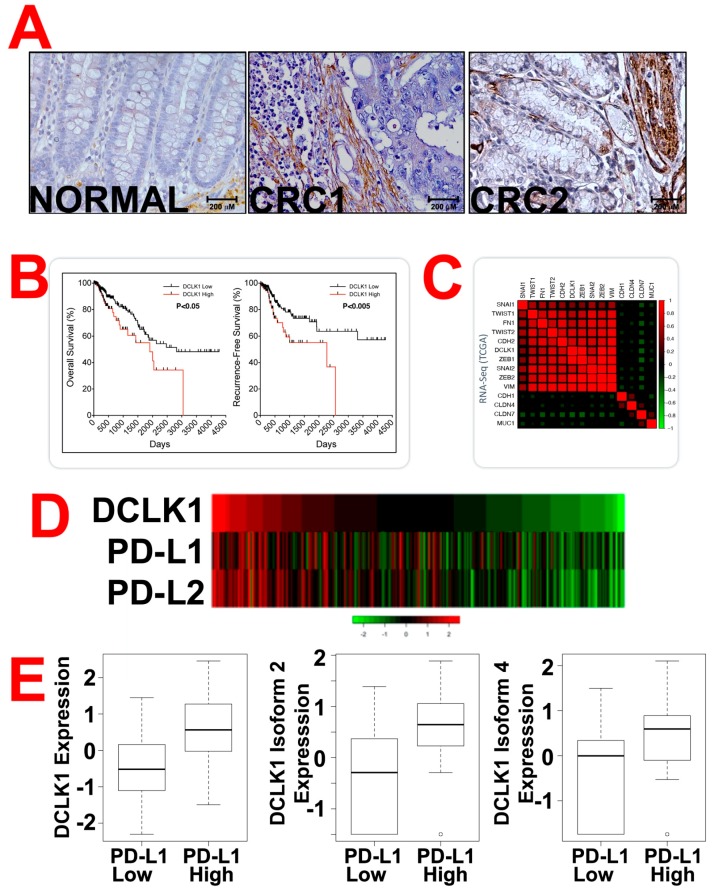
Doublecortin-like kinase 1 (DCLK1) expression in human colorectal cancer patient tissues. (**A**) Human colorectal cancer (CRC) tissues and adjacent normal tissues were subjected to immunohistochemical analyses for DCLK1 using hCBT-15 mAb. Representative images are presented. Scale bars are presented at the image. (**B**) The Cancer Genome Atlas (TCGA) data was generated using Illumina HiSeqV2 RNA-sequencing data and was sorted for overall survival of the patients with low or high DCLK1 expression. (**C**) Moreover, TCGA data was generated and sorted for the expression of various epithelial-mesenchymal transition (EMT)-related transcription factors in the samples with low or high DCLK1 expression indicating that EMT and DCLK1 expression is correlated with poor overall survival. (**D**) Heatmap of the TCGA analyses for DCLK1 and immunomodulating checkpoints proteins programmed cell death-ligand 1 (PD-L1) and PD-L2. (**E**) TCGA data were sorted for samples with low or high PD-L1 expression. The samples with high PD-L1 had increased expression of DCLK1, cancer stem cells (CSCs)-associated DCLK1 isoform 2 and isoform 4 (*p* < 0.01 for all comparisons).

Our previous studies demonstrated that DCLK1 is highly upregulated in human CRCs and that this upregulation is associated with poor clinical outcomes [27]. As part of the validation of CBT-15, we used a tissue microarray containing human CRC tissues and adjacent normal tissues and immunohistochemistry (IHC) to assess levels of DCLK1. DCLK1 expression was increased in human CRC tissues compared to adjacent normal (Figure 2A). This data is relatively consistent with our previous reports using an anti-DCLK1 polyclonal antibody (Abcam, Cambridge, MA, USA, catalog # ab31704) [21,23]. Additionally, we have immunostained several human tissues (normal and cancer) with CBT-15 (DCLK1 mAb). There was increased expression of DCLK1 in human cancers (Kidney, CRC, Liver, Esophagus, Bladder, Cervix, Uterus, Rectal, Lung, Ovary, Melanoma, and Breast) compared to human normal tissues (Supplementary Figure S1). To measure DCLK1 mRNA expression and determine if the levels correlated with patient survival, we analyzed the Illumina HiSeqV2 RNA-sequencing The Cancer Genome Atlas (TCGA) database. We observed a significant increase in overall and recurrence-free survival in patients with low DCLK1 expression and reduced overall and recurrence-free survival in patients with high DCLK1 expression (Figure 2B). Interestingly, patients with high DCLK1 had increasing amounts of EMT-related transcription factors (e.g., SNAI1, TWIST1, ZEB1, ZEB2 and Vimentin; Figure 2C). These data taken together suggest that high DCLK1 is associated with increased EMT transcription factors within the human CRC tissues. Furthermore, increased DCLK1 RNA (isoforms 2 and 4) was associated with increased expression of programmed cell death-ligand 1 (PD-L1) and PD-L2 mRNAs, suggesting that patients with high DCLK1 may be more responsive to immune check point inhibitors (Figure 2D,E; *p* < 0.01).

### 2.3. CBT-511, CAR-T Cells Generated with DCLK1 CBT-15 Antibody ScFv, Recognizes DCLK1 Protein

**Figure 3:**
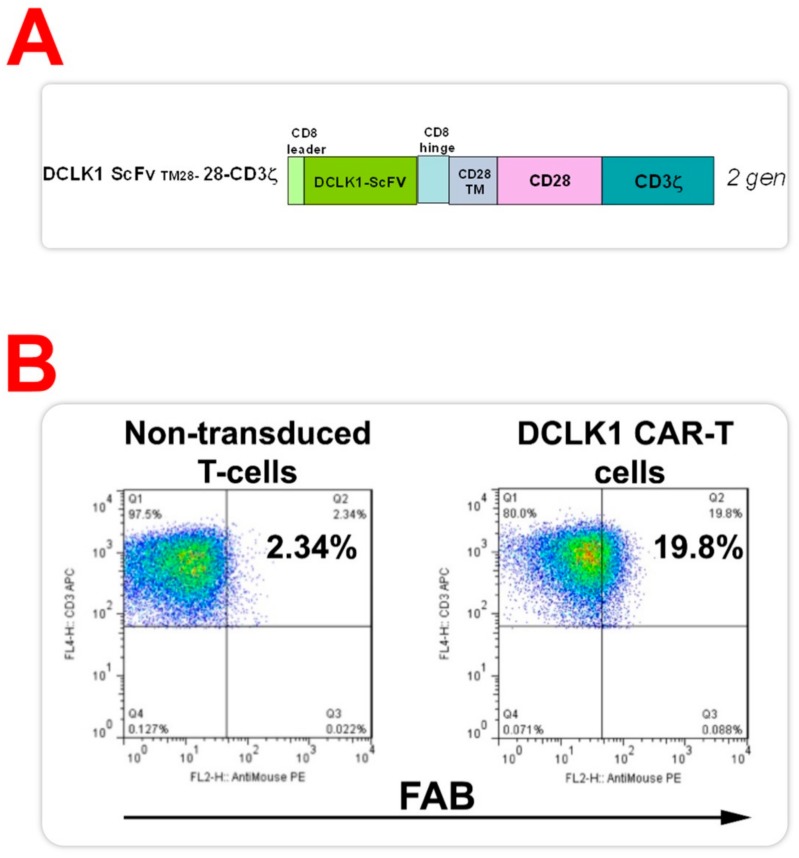
Expression of chimeric antigen receptors (CARs) in Doublecortin-like kinase 1 (DCLK1) CAR-T cells (CBT-511). (**A**) The illustration represents the structure of DCLK1-CAR (CBT-511). The second-generation CAR with DCLK1 ScFv, CD28 co-stimulatory domain, and CD-3zeta activation domain was generated. (**B**) Expression of DCLK1-CAR in T cells transduced with lentiviral DCLK1-CAR by Fluorescence-activated cell sorting (FACS) with F(ab)2 antibody. CBT-511 cells were effectively transduced with DCLK1-CAR and expression of CAR (~20%) was confirmed by FACS with F(ab)2 antibody.

Using the ScFv of the hCBT-15 DCLK1 mAb, we created a second-generation DCLK1-based CAR-T cell (CBT-511; ProMab biotechnologies, Richmond, CA, USA) (Figure 3A) [39,41,42,43]. CBT-511 has DCLK1 ScFv fused to a CD28 transmembrane domain and co-stimulatory domain with a CD8 hinge. CBT-511 also contains a CD3zeta activation domain (Figure 3A). CAR-T cells generated using DCLK1 ScFv expanded in vitro and demonstrated ~20% CAR expression when analyzed by Fluorescence-activated cell sorting (FACS) using F(ab)2 antibody compared to non-transduced T cells (Figure 3B). These data demonstrate that DCLK1 CAR-T cells had sufficient DCLK1 CAR expression and were used in further experiments. 

### 2.4. CBT-511 Effectively Kills CRC Cells and Induces the Secretion of IFN-γ

**Figure 4:**
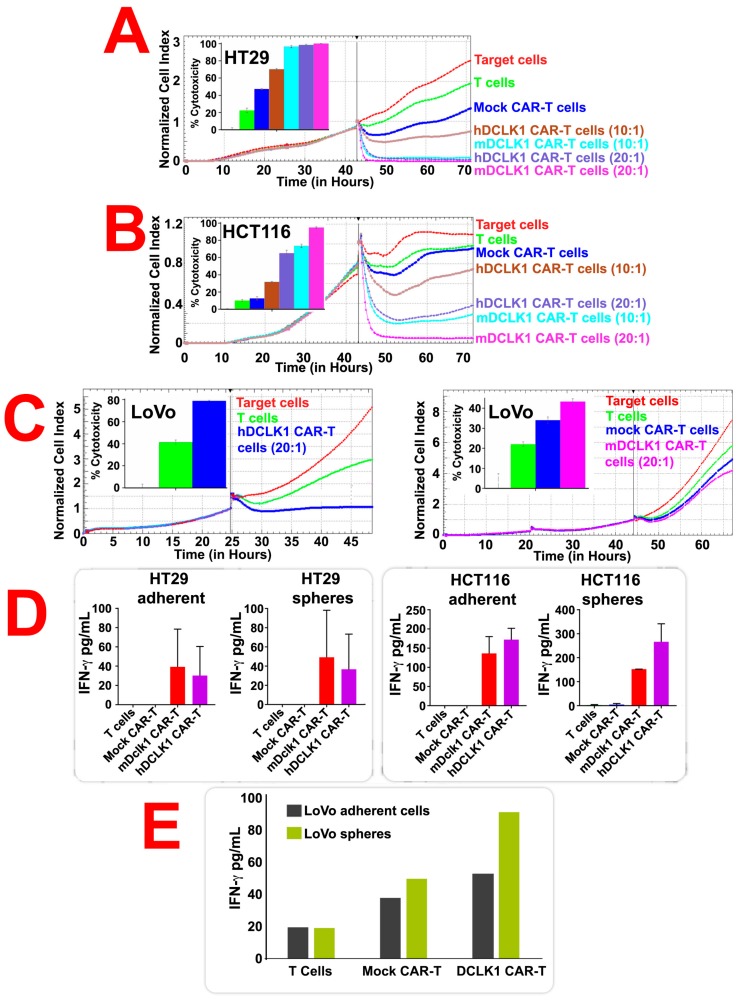
CBT-511 treatment induced colorectal cancer (CRC) cell cytotoxicity and secretion of Interferon gamma (IFN-γ). Human CRC cells (HT29, HCT116, and LoVo) were subjected to real-time cytotoxicity assays (RTCA). Doublecortin-like kinase 1 (DCLK1) CAR-T cells effectively killed HT29 cells compared to mock CAR-T or T cells alone (**A**–**C**). Bar graphs in (**A**,**B**): Cytotoxicity of human DCLK1 (hDCLK1)-CAR-T and mouse Dclk1 (mDCLK1)-CAR-T cells versus T cells and Mock-CAR-T cells. The quantitation of RTCA from three independent experiments is shown. All pairwise comparisons were significant (*p* < 0.0001), except for Mock CAR-T cells versus T cells (*p* = 0.9363) and hDCLK-1-CAR-T cells 20:1 versus mDCLK-1-CAR-T cells 10:1 (*p* = 0.0703). Bar graphs in (**C**): All pairwise comparisons were significant (*p* < 0.05), except for Mock CAR-T cells versus T cells (*p* = 0.2017) and Mock CAR-T cells versus mDclk-1-CAR-T cells (*p* = 0.3769). All p-values were adjusted using Tukey’s method. (**D**) Treatment of HT29 cells grown in 2D with CBT-511 resulted in a dramatic release of IFN-γ (~25 pg/mL) as compared to mock CAR-T cells (0 pg/mL; *p* < 0.01). However, treatment of HT29 cells grown in 3D with CBT-511 resulted in an even higher IFN-γ release (~32 pg/mL versus 0 pg/mL; *p* < 0.01) compared to mock CAR-T. Similar treatment of HCT116 cells with CBT-511 resulted in increased IFN-γ release grown in 2D culture (150 pg/mL) compared to mock CAR-T (0 pg/mL). However, treatment of HCT116 cells grown in 3D with CBT-511 resulted in an even higher IFN-γ release (~250 pg/mL) compare to mock CAR-T (0 pg/mL; *p* < 0.01). (**E**) Treatment of LoVo cells with CBT-511 resulted in increased IFN-γ release grown in 2D culture (~50 pg/mL) compared to mock CAR-T (~30 pg/mL). However, treatment of LoVo cells grown in 3D with CBT-511 resulted in an even higher IFN-γ release (~95 pg/mL) compare to mock CAR-T (50 pg/mL). These data taken together suggests that CBT-511 is more active against CRC cells grown in 3D culture consistent with the increased clonogenic capacity of Tumor stem cells (TSCs).

In real-time cytotoxicity assays (RTCA), CRC cells grown in 2D were co-cultured with effector cells (DCLK1 CAR-T cells or mock CAR-T cells) at dilutions of 10:1 and 20:1 (effector cells: CRC cells). Additionally, based on our previous experience and recent published reports an E:T 10:1 and 20:1 are optimal [39,42,43]. The CRC cells were monitored for another one to two days with the RTCA xCELLigence system (Acea Biosciences, San Diego, CA, USA), and impedance were plotted over time and cytolysis were calculated. At 72h, DCLK1 CAR-T cells (10:1 dilution) effectively killed ~30% of HT29 cells and 23% of HCT116 cells (Figure 4A,B). However, at 20:1 dilution, DCLK1 CAR-T cells killed nearly 100% of HT29 cells, 60% of HCT116, and 78% of LoVo cells (Figure 4A–C). These data suggest that DCLK1 CAR-T cells effectively kill CRC cells grown in 2D at 20:1 dilution. In order to demonstrate the effects of CBT-511 on IFN-γ release, we performed co-culture experiment similar to RTCA. IFN-γ release is the best measure of CAR-T interaction with target cells that results in cytotoxicity of the cancer cells. CAR-T cells were co-cultured with CRC cells grown in 2D culture system and in 3D matrices as spheroids separately. The supernatant from these experiments were collected and subjected to IFN-γ release assay using enzyme-linked immunosorbent assay (ELISA). Treatment of HT29 cells grown in 2D with CBT-511 resulted in a dramatic release of IFN-γ as compared to mock CAR-T cells (~25 versus 0 pg/mL; *p* < 0.01). However, treatment of HT29 cells grown in 3D with CBT-511 resulted in an even higher IFN-γ release (~32 pg/mL versus 0 pg/mL; *p* < 0.01; Figure 4D). Treatment of HCT116 cells grown in 2D with CBT-511 resulted in a dramatic release of IFN-γ as compared to mock CAR-T cells (~150 versus 0 pg/mL; *p* < 0.01). However, treatment of HCT116 cells grown in 3D with CBT-511 resulted in an even higher IFN-γ release (~250 pg/mL versus 0 pg/mL; *p* < 0.01; Figure 4D). In LoVo cells however, we did not observe a similar pattern of IFN-γ release. In 2D, there was ~50 pg/mL of IFN-γ release with CBT-511 treatments; however, in cells treated with mock CAR-T treatments, there was 35 pg/mL of IFN-γ release (Figure 4E). Whereas in 3D, there was ~90 pg/mL of IFN-γ release with CBT-511 treatments and in mock CAR-T treatments there was ~50 pg/mL of IFN-γ release (Figure 4E). These data taken together suggest that although DCLK1 CAR-T cells induced IFN-γ release as expected, the mock CAR-T cells also induced IFN-γ release indicating a non-specific IFN-γ response in LoVo cells. These data suggest that hDCLK1-specific CAR-T cells successful bind to CRC cells and induce IFN-γ secretion consistent with CAR-T cells-induce cytotoxicity.

### 2.5. CBT-511 Blocks Subcutaneous LoVo CRC Cells-Derived Xenograft Tumor Growth In Vivo

**Figure 5:**
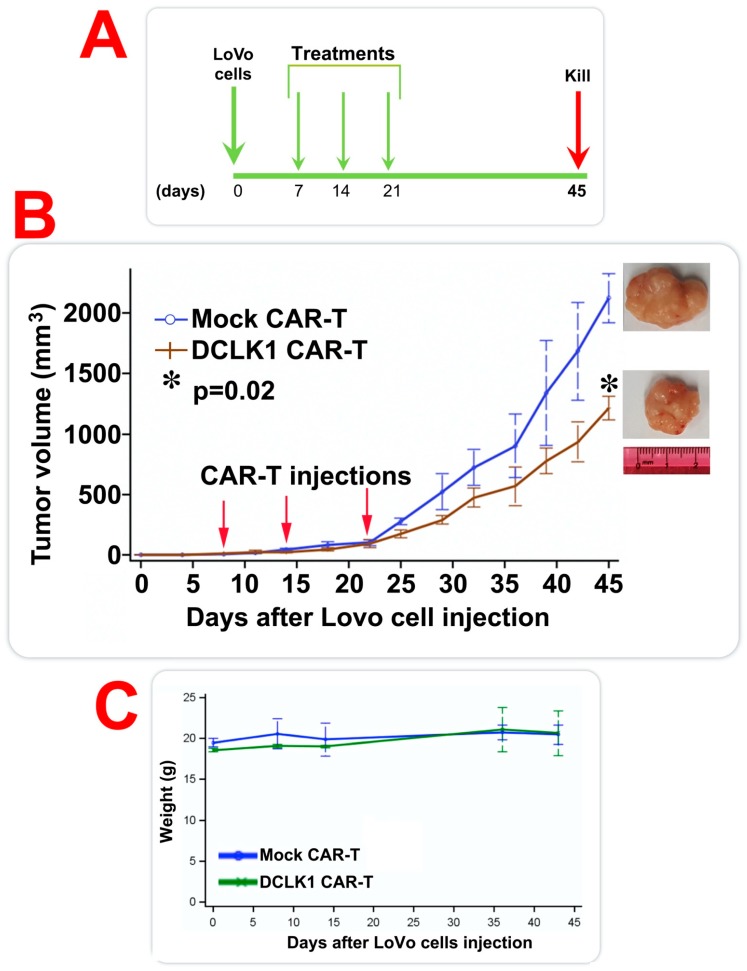
CBT-511 treatment reduced LoVo, human colorectal cancer (CRC) cell-line induced tumor xenograft growth. (**A**) LoVo, human CRC cells were injected subcutaneously into NOD Scid gamma (NSG™) mice and the tumors were allowed to grow. On days 7, 14, and 21 post-cell implantations, the mice were injected intravenous (*i.v.*) with either Doublecortin-like kinase 1 (DCLK1) CAR-T or mock CAR-T cells (1 × 10^7^ cells/mice). Tumor volumes were measured and all the mice were weighted during the experiment period. (**B**) CBT-511 treatment resulted in a significant (* *p* = 0.02) decreased tumor xenograft growth compared to treatment with mock CAR-T cells. The mice treated with CBT-511 had smaller tumors compared to mock CAR-T treated mice. (**C**) There were no significant differences in the animal weights between CBT-511 and mock CAR-T treated mice.

To confirm the in vivo efficacy of DCLK1 CAR-T cells in CRC cell lines, we generated LoVo CRC cell line-derived tumor xenografts in NSG™ mice. Although we did not observe a significant increase in cell surface DCLK1 expression in LoVo cells grown in 3D compared to cells grown in 2D, we observed a 23% extracellular DCLK1 expression at baseline in adherent cells (2D). This level of expression is significantly higher than observed in many cancer cell lines (data not shown) and higher than HT29 cells (9%) in 2D. This may explain the robust cytotoxicity seen in CRC cell lines grown in adherent conditions. We chose to evaluate the effects of the DCLK1 CAR-T in LoVo cells, because this colorectal cancer cell line is derived from the distant metastatic lesion of a 56-year old patient with a histologically confirmed adenocarcinoma of the colon. Furthermore, the cell line has been well characterized and has been used extensively to evaluate the effects of drugs on colony formation and clonogenicity [50]. Moreover, the cell line is reported to have oncogenic activation of cMYC and RAS [51] that have been associated with DCLK1 [22,52]. Tumor-bearing mice were given intravenous (*i.v.*) injections of DCLK1 CAR-T or mock CAR-T cells (1 × 10^7^ cells/mice/dose) every week for three weeks (days 7, 14, and 21 post-cell implantations; Figure 5A). The tumor growth and animal body weights were measured every week until day 45. The mice were euthanized and tumors were extracted and final volumes were measured. Mice treated with CBT-511 had a significant reduction in tumor size compared to those given mock CAR-T treatments (mean tumor volume of mock CAR-T versus DCLK1 CAR-T = 2123 versus 1216 mm^3^ with a *p* = 0.02; Figure 5B). These data taken together indicate that DCLK1-based CAR-T cells when administered *i.v.* significantly inhibited CRC tumor growth. Additionally, there was no significant difference in animal body weights between the CBT-511-treated and mock-CAR-T-treated animals (Figure 5C). Based on this in vivo experiment, these data taken together strongly suggests that DCLK1 CAR-T treatments did not have any obvious toxicity in the mice.

## 3. Discussion

Advanced CRC remains an extraordinarily difficult cancer to treat. CRC is the third-leading cause of cancer death in the U.S. It represents an enormous challenge and has a poor overall survival (~12% 5-year overall survival (OS); Stage IV CRC). Novel therapeutic approaches designed to eradicate metastatic dissemination of CRC cells would fill a major gap in successful therapy. 

Adoptive T cell-based immunotherapy has the potential to improve patient outcomes due to the ability of cytotoxic T cells that target the tumor epithelium, penetrate the TME, and eliminate the tumor. CAR-T cell therapy builds upon this approach by using the patient’s own T cells to activate, proliferate, and destroy tumor cells. This is accomplished by engineering the T cells to express a specific tumor-related antigen that is recognized by the extracellular targeting region of the CAR-T. In this report, we describe a novel CSC-directed CAR-T that specifically binds to the extracellular regions of the TSC protein DCLK1 expressed in CRC. Using LoVo CRC cells derived from metastatic tissue from a patient with adenocarcinoma of the colon, we demonstrate that CAR-T cells expressing the ScFv region of a humanized mAb directed at isoforms 2 and 4 of human DCLK1 (hCBT-15) reduced LoVo colon cancer xenograft growth by greater than 42 percent compared to mock CAR-T controls. This occurred without any weight loss or obvious adverse effect of CAR-T cells compared to controls. 

One major determinant of successful CAR-T therapy is the specificity of the mAb, and as such the ScFv regions used to generate the CAR-T against the cell surface expression of the target antigen. With respect to DCLK1 surface expression, many cancer cell lines express low levels of extra cellular isoforms 2 and 4 (e.g., BxPC3 and MIA PaCa-2 pancreatic cancer cell lines); however, in this report, we describe three cancer cell lines that express greater than 9 percent DCLK1 cell surface expression at baseline: HT29, HCT116, and LoVo cells. This level of expression was sufficient to allow CAR-T cells at 20:1 dilution to exhibit significant real time cytotoxicity. However, our overall hypothesis is that the clonogenic capacity of the CSC population is the true target of successful immunotherapy. When CRC cell lines were subsequently grown in 3D culture systems in order isolate the clonogenic population, we observed a marked increase in the expression of extracellular DCLK1 isoforms in HT29 cells (from 9 to 45%). Interestingly, we did not observe a similar increase in HCT116 or LoVo cancer cells. However, baseline expression in these two cell lines was higher than that observed in the HT29 cell line. We predict that in 3D clonogenic culture systems, we select for more undifferentiated stem-like cells [53]. HCT116 and LoVo cells had relatively higher expression of surface DCLK1 when grown in 2D compared to HT29. Although these findings are under investigation in our laboratory, tumorspheres have more undifferentiated cells that express surface DCLK1 compared to cells grown in 2D. Moreover, in these cell lines, we did not observe an increase in DCLK1 cell surface expression when grown in 3D. We speculate that LoVo cells have a more mesenchymal-like phenotype [54] and may express sufficient DCLK1 to promote tumorigenesis, whereas HT29 cells, which appear to be of a more epithelial phenotype, require a less differentiated state in order to demonstrate increased DCLK1 expression on the surface. LoVo cells, according to recent reports, generally express more vimentin than E-cadherin, suggesting a more mesenchymal phenotype compared to HCT116 or HT29 [54]. Nevertheless, studies are underway to assess the sensitivity of clonogenic cells to CAR-T based cytotoxicity. 

Our in vitro studies using HT29, HCT116, and LoVo cells cultured in 3D matrices revealed a significant increase in IFN-γ release, a surrogate measure of cytotoxicity, compared to cells grown in 2D with a 19.8 percent DCLK1 CAR expression. However, we are fully aware that further studies are needed to fully assess cytokine release and accurate cytotoxicity of the specific clonogenic cells (CSC) within spheroids. In future studies, we intend to reclone the DCLK1 ScFv into a vector containing the MND U3 promoter. In preliminary studies, this promoter resulted in a nearly three-fold increase in cell surface CAR expression compared to the EF1 promoter we used in this report [55]. These data support our overall hypothesis that the clonogenic population of tumor cells may represent an ideal target for DCLK1 driven CAR-T therapy. Because DCLK1 cell surface expression is increased on a diverse range of TSCs, the need for highly specific CAR-T cells may be reduced. It is important for us to emphasize that unlike traditional extracellular antigens that are upregulated in tumors but undetectable in normal tissues, DCLK1 is highly upregulated in tumor cells and expressed at low levels in normal tissues. However, the specific TSC population and progeny are the targets of DCLK1 CAR-T therapy. Furthermore, because DCLK1 is not expressed on normal stem cells, the risk for off-target serious adverse effects may be reduced [25]. One particular advantage of a DCLK1-based anti-TSC strategy is that although DCLK1 marks TSCs, it is not expressed on normal LGR5^+^ stem cells Thus, DCLK1 is the first and only TSC marker that can distinguish between normal cells from TSCs [25].

Analysis of the TCGA database of patients with CRC revealed that elevated levels of DCLK1 mRNA transcripts compared to normal adjacent tissue was associated with a poor prognosis and reduced OS. Conversely, patients with low DCLK1 expression levels exhibited an increase in overall and recurrence-free survival. At the protein level, tissue microarray of patients with CRC had increased levels of DCLK1 compared to normal adjacent tissues. These data illustrate the potential importance of targeting DCLK1 to eliminate and perhaps inactivate the TSC population in CRC [19,21,22,23,24].

Low levels of DCLK1 are associated with increased OS and recurrence free survival and are associated with low expression of EMT transcription factors. These data suggest that reduction of DCLK1 and DCLK1 expressing cells may be an important goal of CRC therapies aimed at reducing EMT factors to improve OS outcomes [19,21,22,23,24]. We previously demonstrated that interruption of DCLK1 signaling blocked EMT and inhibited the invasion and migration of cancer cells [20,22,23,24]. Furthermore, siRNA-mediated knockdown of DCLK1 has been shown to upregulate anti EMT, miR-200 family of microRNAs with downregulation of transcription factors—ZEB1, SNAIL and SLUG [19,20,21,22].

A major study by Maliar et al., demonstrated that a CAR-T construct targeting the putative CSC protein CD24 in pancreatic ductal adenocarcinoma (PDAC) improved overall survival (OS) and reduced metastasis in orthotopic patient-derived xenografts and in CAPAN-1-derived xenografts. In separate experiments, treatment with Her2/neu CAR-T cells also resulted in improved OS, and tumor eradication in xenograft models [56]. However, the anti Her2/neu CAR-T cells only demonstrated significant efficacy when epidermal growth factor receptor (EGFR) was highly expressed in the majority of cells within the tumor, whereas anti-CD24 CAR-T was successful despite low levels of CD-24 expression within the tumor. These data taken together illustrate that targeting CSCs can display similar efficacies to CAR-Ts targeting antigens expressed on rapidly proliferating tumor cell populations. The studies presented above support our rationale for targeting a highly specific TSC antigen for the treatment of CRC and perhaps other solid tumors.

## 4. Materials and Methods 

### 4.1. Cells, Primary Tissues

CRC cell lines HT29, HCT116, and LoVo were purchased from the ATCC (Manassas, VA, USA) and cultured in RPMI-1640 medium (ThermoFisher, Waltham, MA, USA) containing 10% Fetal Bovine Serum (FBS) (AmCell, Mountain View, CA, USA). Human peripheral blood mononuclear cells (PBMC) were isolated from whole blood obtained in the Stanford Hospital Blood Center (Stanford University) according to Institutional Review Board (IRB)-approved protocol (#13942). PBMC were isolated by density sedimentation over Ficoll-Paque (GE Healthcare, Chicago, IL, USA).

### 4.2. Monoclonal Antibody

DCLK1-targeted monoclonal antibody (CBT-15 mAb), DCLK1 mAb analogue (previously reported, see [47,48]), and isotype control mAb were generated as previously described. Binding affinity of CBT-15 was confirmed by enzyme-linked immunosorbent assay (ELISA) using full-length DCLK1 [47,48,57].

### 4.3. Generation of CAR-Encoding Lentivirus

DNA encoding the DCLK1 CAR was synthesized and subcloned into a third-generation lentiviral vector, Lenti CMV-MCS-EF1a-puro by Syno Biological (Beijing, China) [39,42]. Ten million growth-arrested HEK293FT cells (ThermoFisher) were seeded into T75 flasks. Cells were cultured overnight and transfected using the CalPhos Transfection Kit (Takara, Mountain View, CA, USA) and pPACKH1 Lentivector Packaging Mix (System Biosciences, Palo Alto, CA, USA) containing 10 µg of the lentiviral vector. The media was changed the following day and 48 h later, media containing the lentivirus was collected and clarified by centrifugation at 2100× *g* for 30 min. The virus particles were collected by centrifugation at 112,000× *g* for 100 min, suspended in AIM V medium, aliquoted, and frozen at −80 °C. The titers of the virus preparations were determined by quantitative Reverse transcription polymerase chain reaction (RT-PCR)using the Lenti-X qRT-PCR kit (Takara) according to the manufacturer’s protocol and the 7900HT thermal cycler (ThermoFisher). The lentiviral titers were >1 × 10^8^ plaque forming units (pfu)/mL [39,42].

### 4.4. Generation and Expansion of CAR-T Cells

PBMCs were suspended at 1 × 10^6^ cells/mL in AIM V-AlbuMAX medium (ThermoFisher) containing 10% FBS and 10 ng/mL IL-2 (ThermoFisher), mixed with an equal number (1:1 ratio) of CD3/CD28 Dynabeads (ThermoFisher), and cultured in non-treated 24-well plates (0.5 mL per well) [39,42]. At 24 and 48 h, lentivirus was added to the cultures at a multiplicity of infection (MOI) of 5 along with 1 µL of TransPlus transduction enhancer (AlStem, San Francisco, CA, USA). As the T cells proliferated over the next 10–12 days, the cells were counted every 2–3 days and spent media was replaced with fresh media containing 10 ng/mL IL-2 was added to maintain the cell density at 1 to 3 × 10^6^ cells/mL [39,42].

### 4.5. Flow Cytometry

To measure CAR expression, 0.25 million cells were suspended in 100 µL of buffer (Phosphate-buffered saline (PBS) containing 2 mM Ethylenediaminetetraacetic acid (EDTA) pH 8 and 0.5% Bovine serum albumin (BSA)) and incubated on ice with 1 µL of human serum (Jackson Immunoresearch, West Grove, PA, USA) for 10 min. Then, 0.3 µg of biotinylated human DCLK1 protein (AcroBiosystems, Newark, DE, USA) was added and the cells were incubated on ice for 30 min. Cells were rinsed with 3 mL of buffer and suspended in 100 µL of buffer. Next, 1 µL of phycoerythrin (PE)-conjugated streptavidin (BD Biosciences, San Jose, CA, USA), 1 µL of allophycocyanin (APC)-labeled anti-CD3 (BioLegend, San Diego, CA, USA) and 2 µL of 7-aminoactinomycin D (7-AAD) solution (BioLegend) were added to the cells and incubated on ice for 30 min [39,42]. The cells were rinsed with 3 mL of buffer and suspended in buffer and acquired on a FACSCalibur (BD Biosciences). Cells were analyzed first for light scatter versus 7-AAD staining, and the 7-AAD^−^ live gated cells were plotted for anti-CD3 staining versus DCLK1 protein staining [39,42].

### 4.6. Cytokine Induction Assay

Target cells (HT29, HCT116, and LoVo) were cultured with the effector cells (CAR-T cells or non-transduced T cells) at a 10:1 ratio (E:T; 1 × 10^4^ cells each) in U-bottom 96-well plates with 200 µL of AIM V-AlbuMAX medium containing 10% FBS, in triplicate. After 16 h, the top 150 µL of medium was transferred to V-bottom 96-well plates and centrifuged at 300 × g for 5 min to pellet any residual cells. The top 120 µL of supernatant was transferred to a new 96-well plate and analyzed by ELISA for human IFN-γ levels using a kit from R&D Systems (Minneapolis, MN, USA) according to the manufacturer’s protocol. Additionally, based on our previous experience and published report an E:T 10:1 and 20:1 are optimal for cytokine induction and cytotoxicity [39,42,43].

### 4.7. Real-Time Cytotoxicity Assay (RTCA)

Adherent target cells (HT29, HCT116, and LoVo) were seeded into 96-well E-plates (Acea Biosciences, San Diego, CA, USA) at 1 × 10^4^ cells per well and monitored in culture overnight using impedance-based real-time cell analysis (RTCA) xCELLigence system (Acea Bio). The next day, the media was removed and replaced with AIM V-AlbuMAX medium containing 10% FBS ± 1 × 10^5^ effector cells (CAR-T cells or non-transduced T cells), in triplicate. The cells in the E-plates were monitored for another 24–48 h with the RTCA system, and impedance was plotted over time. Cytolysis was calculated as follows: (impedance of target cells without effector cells-impedance of target cells with effector cells) × 100/impedance of target cells without effector cells.

### 4.8. Mouse Xenograft Study

Six-week old male NSG™ mice (Jackson Laboratories, Bar Harbor, ME, USA) were housed and manipulated in strict accordance with the Institutional Animal Care and Use Committee (IACUC). All animal experiments were performed at Joint Innovation Park Animal Facility, Richmond, CA according to approved IACUC protocol. Each mouse was injected subcutaneously (both sides of the flanks; *n* = 3 mice with six tumors in each group) on day 0 with 100 μL of 1 × 10^7^ LoVo cells/mice separately. CAR-T cells (CBT-511 or mock CAR-T cells) were injected intravenously (1 × 10^7^ cells/mice each day) on days 7 or 14 and 21. Tumor sizes were measured with calipers twice weekly and tumor volume (in mm^3^) was determined using the formula W^2^L/2, where W is tumor width and L is tumor length until day 45. At the end of the studies, tumors were excised and final volumes measured.

### 4.9. Statistical Analysis

Data were analyzed and plotted using Prism software (GraphPad, San Diego, CA, USA). Comparisons between two groups were performed by unpaired Student’s t-test. Comparisons between three or more groups were performed by one-way ANOVA or two-way ANOVA followed by Tukey’s or Sidak’s post hoc test. The difference with *p* < 0.05 was considered significant.

## 5. Conclusions

Together, these results indicate that DCLK1-targeted CAR-T immunotherapy provides a novel mechanism for eliminating the cell of origin and perhaps the metastatic progeny in CRC. This study is the first of its kind to demonstrate the therapeutic utility of a CAR-T directed against a TSC-specific antigen for the treatment of CRC. The results of this study may lead to the development of novel TSC-directed CAR-T therapies for CRC and perhaps other solid tumor cancers.

## 6. Patents

This work is part of a provisional patent application filed by COARE Biotechnology Inc.

## Supplementary Materials

The following are available online at https://www.mdpi.com/2072-6694/12/1/54/s1, Figure S1: DCLK1 expression in human tissues.
