# Supplementary material for: DCLK1 Monoclonal Antibody-Based CAR-T Cells as a Novel Treatment Strategy against Human Colorectal Cancers
Source: Cancers (Basel). 2019 Dec 23;12(1):54. doi: 10.3390/cancers12010054 (PMC7016951; doi:10.3390/cancers12010054)
Supplement: Supplementary file 1 [file cancers-12-00054-s001.pdf]

Supplemetray Materilas

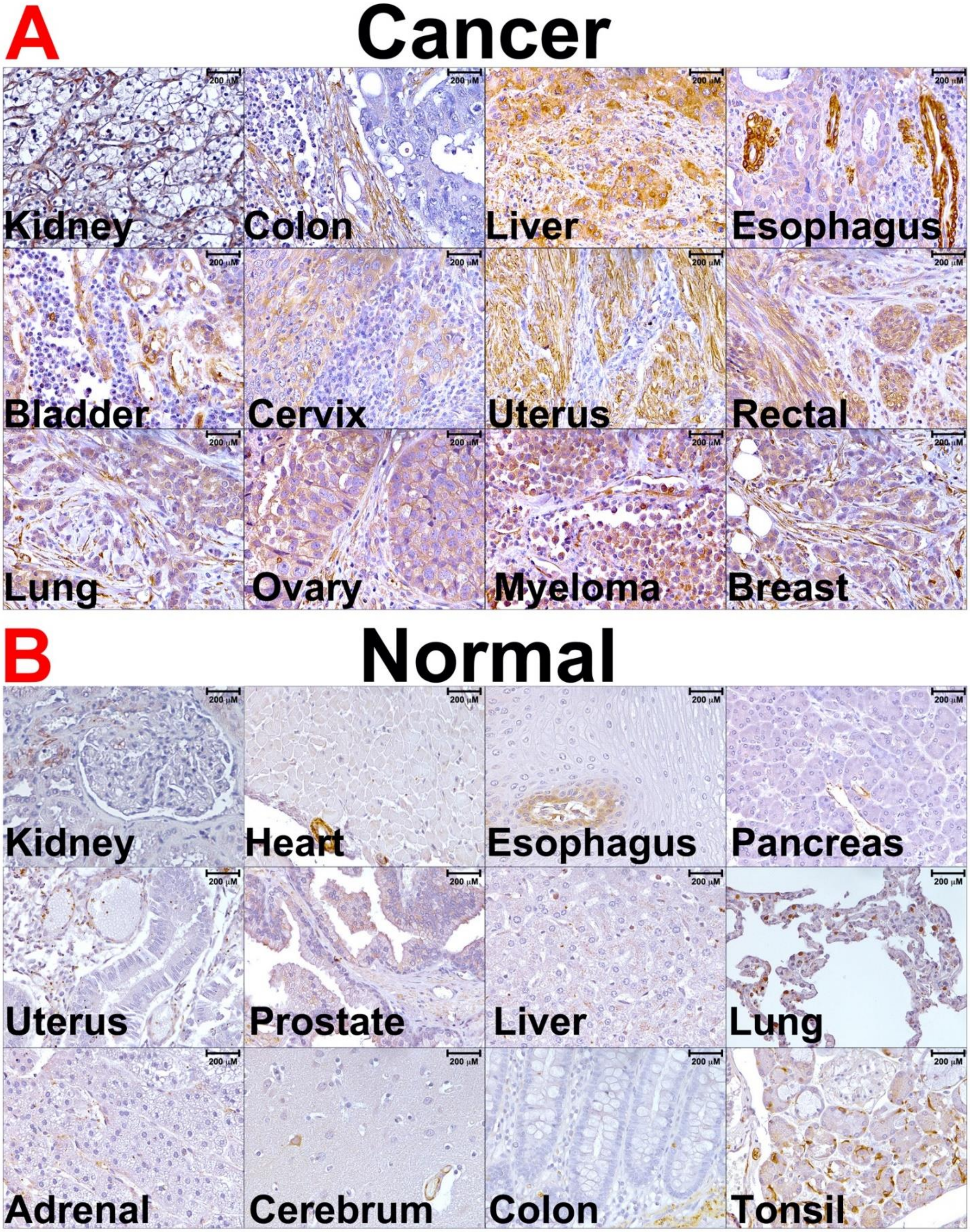

Figure S1. DCLK1 expression in human tissues. Human cancer tissues and normal tissues were subjected to immunohistochemical analyses for DCLK1(brown) using hCBT-15 mAb. Representative images are presented. Human cancer tissues (Kidney, CRC, Liver, Esophagus, Bladder, Cervix, Uterus, Rectal, Lung, Ovary, Melanoma, and Breast) expressed increased DCLK1 (**A**) compared to human normal tissues (Kidney, Heart, Esophagus, Pancreas, Uterus, Prostate, Liver, Lung, Adrenal, Cerebrum, Colon, and Tonsil) (**B**). Scale bars are presented at image.
